# Long-Term Outcomes of Cervical Laminoplasty in the Elderly

**DOI:** 10.1155/2015/713952

**Published:** 2015-10-25

**Authors:** Yasushi Oshima, Kota Miyoshi, Yoji Mikami, Hideki Nakamoto, Sakae Tanaka

**Affiliations:** ^1^Department of Orthopaedic Surgery, The University of Tokyo, 7-3-1 Hongo, Bunkyo-ku, Tokyo 113-8655, Japan; ^2^Department of Orthopaedic Surgery, Yokohama Rosai Hospital, 3211 Kodukuecho, Kohoku-ku, Yokohama, Kanagawa 222-0036, Japan

## Abstract

Incidences of cervical laminoplasty in the elderly are increasing; the influence of other age-related complications and neurological status must be considered for justifying surgery. This study identified the aforementioned influence on long-term outcomes of cervical laminoplasty in patients aged ≥75 years. Thirty-seven of 38 consecutive patients aged ≥75 years who underwent cervical laminoplasty were retrospectively evaluated. Minimum 5-year follow-up was acceptable if patients were complication-free. Follow-up was terminated when neurological evaluation was not possible, owing to death or other serious complications affecting activities of daily living (ADL). Postoperative neurological changes and newly developed severe complications were investigated. Postoperatively, one patient died of acute pneumonia, one remained nonambulatory owing to cerebral infarction, and 35 were ambulatory and were discharged. At a mean follow-up of 78 months, three patients died and nine developed serious complications severely affecting ADL. Of the 25 remaining patients, 23 remained ambulatory at mean follow-up of 105 months. Cox proportional hazard analysis revealed that postoperative motor upper and lower extremities JOA scores of ≤2 and ≤1, respectively, were risk factors for mortality or other severe complications. Postoperative neurological status can be maintained in the elderly if they remain complication-free. Poorer neurological status significantly affected their ADL and mortality.

## 1. Introduction

Laminoplasty, a motion preservation surgical procedure for cervical myelopathy, has become popular as a safe and effective treatment for cervical myelopathy with few reported complications and relatively good long-term outcomes [1–6]. Laminoplasty can be employed in patients with multiple-level spinal cord compression as it is suitable for elderly patients with cervical compression myelopathy [7, 8]. Further, this technique is particularly suitable for elderly patients because the incidence of airway complications is less than that occurring in anterior surgery [9, 10]. As the population continues to age, spine surgeons are expected to encounter an increased number of patients with cervical compression myelopathy. Although the neurological recovery rate (RR) in elderly patients may be inferior to that in younger patients, significant clinical improvement shortly after cervical decompression surgeries has been reported in previous reports as well [7, 11–15]. However, long-term outcomes of cervical decompression surgery in elderly patients remain uncertain.

One possible problem in interpreting long-term surgical outcomes in elderly patients is the consideration of the influence of other age-related diseases, such as cerebral infarction, dementia, and severe osteoarthritis. As patients age, the prevalence of such comorbidities increases, complicating the neurological evaluation of cervical myelopathy. Furthermore, patients with severe complications tend to discontinue outpatient treatment because they may no longer visit the hospital; thus, only patients with relatively better activities of daily living (ADL) are typically enrolled in this type of a study. Therefore, a higher follow-up rate is required for accurately evaluating long-term postoperative status, particularly among elderly patients.

Here, we report the long-term prognostic course of elderly patients after cervical laminoplasty, with a follow-up rate of 97%. This study aimed to investigate the long-term outcomes of cervical laminoplasty in terms of the neurological status and age-related comorbidities among patients aged ≥75 years.

## 2. Materials and Methods

We retrospectively reviewed medical records of 235 consecutive patients who underwent laminoplasty for cervical compression myelopathy owing to cervical spondylotic myelopathy (CSM) or ossification of the longitudinal ligament (OPLL) between 1998 and 2004 and identified 38 patients, with a mean age of 78 (75–86) years, at the time of surgery. Patients with rheumatoid arthritis, disc herniation, tumor, trauma, or previous surgery were not included. The study protocols were approved by the institutional review board of the authors' institution. Cervical laminoplasty was performed using autograft bones as spacers. Of these, one patient was excluded because he dropped out as an outpatient within 6 months for unknown reasons. Therefore, 37 patients (97%; 23 men and 14 women; Table 1) for whom the final neurological status or ADL could be investigated were included in this study. The underlying diseases in this cohort comprised CSM (*n* = 30) and OPLL (*n* = 7). Follow-up was terminated when a neurological evaluation was no longer possible, owing to patients' death or other serious complications affecting ADL. All patients underwent thorough cardiac and pulmonary function examinations before surgery. Moreover, postoperative neurological status and complications were investigated. The Japanese Orthopaedic Association (JOA) score was used for assessing the neurological ability:

1. Upper extremity motor function:
  0: impossible to eat with chopsticks or spoon.
  1: possible to eat with spoon but not with chopsticks.
  2: possible to eat with chopsticks, but to a limited degree.
  3: possible to eat with chopsticks, awkward.
  4: no disability.
2. Lower extremity motor function:
  0: cannot walk.
  1: needs cane or aid on flat ground.
  2: needs cane or aid only on stairs.
  3: can walk without cane or aid but slowly.
  4: no disability.
3. Sensory function:
  - Upper extremity.
    - apparent sensory loss.
    - minimal sensory loss.
    - normal.
  - Upper extremity (same as A).
  - Trunk (same as A).
4. Bladder function:
  0: complete retention.
  1: severe disturbance.
  2: mild disturbance.
  3: normal.

RR based on the JOA score was evaluated using a previously described formula [16]:

(1) $$\begin{array}{c} RR=\left[\frac{\left(\text{postoperative JOA score}-\text{preoperative JOA score}\right)}{\left(17-\text{preoperative JOA score}\right)}\times 100\%\right]. \end{array}$$

### 2.1. Statistical Analysis

SPSS 18 (SPSS, Inc., Chicago, IL, USA) was used for all statistical analyses, and a probability (*p*) value of <0.05 was considered significant. Nonparametric analyses were performed using the Mann-Whitney* U* test. Categorical variables were analyzed using the chi-square test. Univariate Cox proportional hazard analysis was used for identifying relevant risk factors. Kaplan-Meier survival analysis was used for evaluating the postoperative period without serious complications.

## 3. Results

We aimed for a minimum 5-year follow-up as long as the patient had no serious complications. During postoperative hospitalization, one patient died of acute pneumonia, while one remained nonambulatory even after surgery. The remaining 35 patients remained ambulatory and were eventually discharged (Table 2). C5 palsy with an MMT grade of 2 occurred in two patients within 2 weeks after surgery, which spontaneously improved in three months. The mean pre- and postoperative total JOA scores at 6 months of the 36 surviving patients were 9.3 and 10.9, respectively, with a mean JOA score RR of 21% (as shown in the Japanese Orthopaedic Association score). The postoperative motor JOA scores of the upper extremities (U/E) (preoperative, 2.0, versus postoperative, 2.6; *p* < 0.001) and lower extremities (L/E) (preoperative, 1.2, versus postoperative, 1.9; *p* < 0.001) were significantly high. Six months after surgery, 51% and 49% of patients revealed improvement of at least one level in the U/E or L/E JOA scores.

During the postoperative follow-up of two years, one patient died and two patients developed severe dementia. The mean pre- and postoperative total JOA scores at 2 years of the 34 patients whose neurological examination was possible were 9.3 and 11.0, respectively, with a mean JOA score RR of 19%. Pre- and postoperative radiographic examinations were performed for the surviving 36 patients. The mean pre- and postoperative C2–7 lateral Cobb angles were 16 degrees (range: −3–37 degrees) and 14 degrees (range: −10–41), respectively (*p* = 0.50). Similarly, the mean pre- and postoperative range of motion were 32 (range: 6–47) and 21 (range: 7–52), respectively (*p* = 0.001).

The neurological status of 29 patients could be evaluated 5 years postoperatively; however, it could not be determined in eight patients, owing to the death of two patients, severe dementia in five patients, and severe Parkinsonism in one patient. The mean pre- and postoperative total JOA scores at 5 years of the 29 patients whose neurological examination was possible were 9.4 and 10.6, respectively, with a mean JOA score RR of 15%. Finally, at a mean follow-up of 78 (0–140) months, three patients died and nine developed serious complications that severely affected ADL (Table 3). Therefore, of the surviving patients, 25 (68%) had no other serious complications, and 23 (92%) of them remained ambulatory at a mean follow-up of 105 (60–140) months.

**Figure 1:**
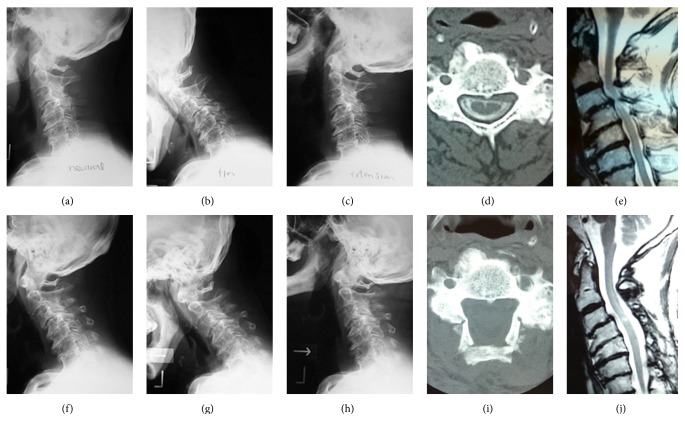
A 76-year-old man who underwent C3–7 laminoplasty for cervical spondylotic myelopathy. Pre- (a–e) and postoperative (f–j) radiographs, CT, and MRI images are shown. Pre/postoperative C2/7 Cobb angles and range of motion were 0/−7 and 33/25 degrees, respectively. Although slight kyphosis progressed after surgery, the patient underwent a good clinical course. Pre- and postoperative (96 months) JOA scores were 9 and 11, respectively.

**Figure 2:**
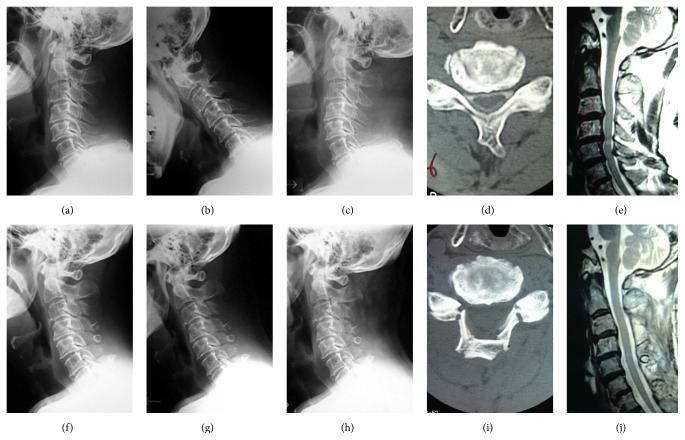
A 79-year-old man who underwent C4–7 laminoplasty for cervical spondylotic myelopathy. Pre- (a–e) and postoperative (f–j) radiographs, CT, and MRI images are shown. Pre/postoperative C2/7 Cobb angles and range of motion were 17/15 and 46/32 degrees, respectively. This patient was not able to walk before surgery but got managed to walk after surgery. However, he developed severe dementia and died 4 years after surgery. Pre- and postoperative (24 months) JOA scores were 5 and 7, respectively.

**Figure 3:**
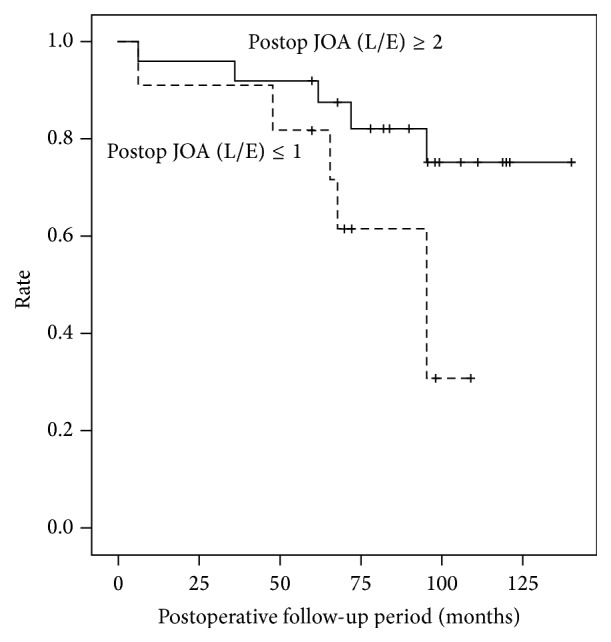
Kaplan-Meier analysis showing the rate of surviving patients without severe complications.

We compared the outcomes of 25 patients for whom a neurological examination was possible (Figure 1) with the 12 patients who died or developed severe complications during follow-up (Figure 2) and found significant differences in postoperative (6 months) motor U/E JOA scores and both pre- and postoperative (6 months) motor L/E JOA scores (Table 4). Furthermore, Cox proportional hazard analysis revealed that a postoperative motor U/E JOA score of ≤2 (hazard ratio (HR): 5.64) and a motor L/E score of ≤1 (HR: 3.47) were risk factors for mortality or other serious complications over a long period (Table 5). According to Kaplan-Meier analysis, 82% of patients with a postoperative L/E JOA score of ≥2 survived and had no severe complications at a mean postoperative follow-up of 78 months, while the rate decreased to 61% among those with a postoperative (6 months) L/E JOA score of ≤1 (Figure 3).

## 4. Discussion

We aimed to clarify factors associated with long-term prognosis after laminoplasty in elderly patients and found that neurological function can be maintained for prolonged periods, unless patients develop severe complications. In contrast, 12 of the 37 patients developed severe comorbidities or died during follow-up, while poorer neurological status significantly affected ADL and mortality.

Several previous reports have indicated that outcomes of cervical laminoplasty in elderly patients may be inferior to those in younger patients [3, 11, 14, 17–19], while others found comparable surgical outcomes [7, 13, 15]. However, the definition of the term “elderly” varied from ≥65 years to ≥80 years, which may explain the inconsistencies in the reported surgical outcomes. As life expectancy has surpassed 80 years in many developed countries, patients aged 65 years are not necessarily considered elderly. Therefore, we defined elderly patients as ≥75 years.

Several studies have reported the surgical outcomes of cervical laminoplasty among an elderly population of ≥75 years. Of these, Matsuda et al. [14] have reported that the JOA score RR was inferior in 17 patients aged >75 years at a mean follow-up of 56 months. In contrast, Nagashima et al. [15] have investigated 37 patients with cervical spondylotic myelopathy aged ≥80 years at a mean follow-up of 15.9 months and reported results similar to those in younger patients. Moreover, Machino et al. [13] have reported that surgical outcomes of 90 patients with cervical myelopathy aged ≥75 years at a mean follow-up of 29 months were as good as those reported in younger patients. Although surgical outcomes of cervical laminoplasty in elderly patients remain controversial, almost all previous studies have shown positive surgical outcomes in elderly patients, at least to some extent, with varying improvement in the JOA recovery rates. However, follow-up was not very long, and the follow-up rate was not very high or unknown. The patients in this study revealed a follow-up rate of 97%, with a mean follow-up of 78 months. Moreover, we could assess the neurological status at a mean follow-up of 105 months, with the exception of patients who developed severe complications or died, which is a considerably long period among elderly patients. To our knowledge, this study reports the longest follow-up in this population.

One possible problem associated with the use of the JOA score RR is that it can be affected by the preoperative JOA score [7, 13, 20]. Instead, the use of the achieved JOA scores may be more suitable for adequately evaluating the surgical effect in elderly patients as the preoperative JOA score is generally lower in this population. As a motor L/E JOA score of 1 is believed to be satisfactory in elderly patients, we should focus on maintaining a long-term ambulatory ability. Hence, we focused on motor JOA scores, particularly those of L/E, instead of the total JOA score RR. In the present study, 92% of patients without severe complications during the postoperative follow-up period remained ambulatory at a mean follow-up of 105 (60–140) months, indicating that the neurological status was also well maintained over a relatively long period.

It is reasonable to speculate that the ambulatory ability will significantly influence mortality and ADL. Indeed, the motor L/E JOA scores significantly affected the incidence of mortality and severe complications. Considering that postoperative neurological status will gradually deteriorate in the future, patients should consider surgical intervention while motor functions are maintained as long as their preoperative comorbidities are not severe. Jiang et al. [5] have reported that the duration of symptoms and severity of stenosis significantly affected surgical outcomes of laminoplasty in elderly patients, supporting the importance of early surgical intervention in elderly patients before irreversible changes to the spinal cord develop. Once patients become bedridden, they will suffer from such complications as pneumonia and coronary diseases. To reduce the risk for becoming bedridden, the importance of physical therapy has been advocated. Recently, the relationship between locomotive functions and health-related quality of life (HRQOL) has been shown [21, 22]. Spreading the need for checking locomotive function in the elderly will not only lead to early detection of myelopathy and osteoarthritis but also reduce the risk for becoming bedridden and severe complications.

During the perioperative period, no patient showed severe neurological deterioration, although two patients suffered C5 palsy. One concern regarding surgical treatment among elderly patients is the higher prevalence of complications. In this study, patients with serious preoperative comorbidities were not considered for surgery; thus, the complication rate was comparable with that in previous reports. Although most complications were temporary, we should consider that one patient died of acute pneumonia shortly after surgery.

Finally, the relationship between poorer neurological status and complications is not limited to the elderly, although poor ADL will lead to severe complications and death more often in the elderly. Indeed, the influences of comorbidity on surgical outcomes are also reported, as measured by such indices as the Charlson Comorbidity Index [23] and the Self-Administered Comorbidity Questionnaire [24]. We may predict adverse events and postoperative HRQOL by using these indices before surgical intervention.

There are several limitations to this study. First, there was no control group. Second, the number of enrolled patients was rather small. Third, no patient-reported outcomes were utilized. Nevertheless, this study had a long follow-up period with a mean of 77 months, which we believe is considerably long in a cohort aged ≥75 years. Because severe complications after cervical laminoplasty are less frequent in younger populations, we feel it would not be informative to compare the prognostic course of elderly patients to those of younger patients.

In conclusion, postoperative neurological status can be maintained over a long period in elderly patients who do not develop severe complications. However, as postoperative neurological status will gradually worsen and a poorer postoperative course will lead to serious comorbidities in the future, we propose that patients without serious preoperative complications should undergo surgical intervention before the neurological status worsens, regardless of age.

## Figures and Tables

**Table 1:** Demographic data of patients.

Number of patients	37
Age (years)	79 (75–86)
Sex (male/female)	22/15
Mean follow-up (months)	78 (0–140)
JOA score	
Preoperative^*∗*^	9.3 (4–14)
Postoperative^*∗*^	10.9 (4–15)
Recovery rate^*∗*^	21% (−67 to 78)

^*∗*^Six months after surgery, excluding one patient who died 2 weeks after surgery.

**Table 2:** Perioperative complications.

Death (pneumonia)	1
Transient ischemic attack	1
Acute cardiac failure	1
Uncontrollable high blood pressure	1
Urinary tract infection	2
Delirium	4
Severe depression	1
Surgical site infection	1
C5 palsy	2
Total	14 (35%)

**Table 3:** Postoperative course and complications.

	Preop	Postop			
		6 months	24 months	60 months	Final (105 months)
Pts. without severe complications	37 (100%)	36 (97%)	34 (92%)	29 (78%)	25 (68%)
Ambulating	29	35	32	27	23
Nonambulating	8	1	2	2	2
Pts. with severe complications (including death)	0 (0%)	1 (3%)	3 (8%)	8 (22%)	12 (32%)
Death	0	1	1	2	3
Dementia	0	0	2	5	5
Parkinsonism	0	0	0	1	1
Schizophrenia	0	0	0	0	1
Cerebral infarction	0	0	0	0	1
Subarachnoid hemorrhage	0	0	0	0	1

**Table 4:** Comparison between patients with or without severe complications at the final follow-up period (average 105 months).

Baseline factors	Without severe complications *n* = 25	With severe complications or dead *n* = 12	*p* value
Age at surgery	76.9 ± 2.2	78.4 ± 3.6	0.20
Sex (male : female)	18 : 7	5 : 7	0.15
Diagnosis (CSM : OPLL)	21 : 4	9 : 3	0.66
Preop C2–7 Cobb angles^*∗*^	18 ± 8	16 ± 10	0.53
Postop C2–7 Cobb angles^*∗*^	15 ± 12	12 ± 9	0.39
Preop ROM^*∗*^	33 ± 11	31 ± 11	0.69
Postop ROM^*∗*^	20 ± 10	21 ± 16	0.59
Pretotal JOA^*∗∗*^	9.5 ± 2.8	8.5 ± 3.3	0.39
Posttotal JOA^*∗∗*^	11.4 ± 2.7	9.9 ± 2.7	0.14
Premotor JOA (U/E)	2.1 ± 0.9	1.7 ± 0.9	0.20
Postmotor JOA (U/E)^*∗∗*^	2.8 ± 0.8	2.2 ± 0.5	0.02
Premotor JOA (L/E)	1.4 ± 0.9	0.8 ± 0.7	0.03
Postmotor JOA (L/E)^*∗∗*^	2.1 ± 0.7	1.4 ± 0.8	0.02

CSM: cervical spondylotic myelopathy; OPLL: ossification of the longitudinal ligament; Preop: preoperative; Postop: postoperative; ROM: range of motion; U/E: upper extremities; L/E: lower extremities.

^*∗*^Two years after surgery, excluding one patient who died 2 weeks after surgery.

^*∗∗*^Six months after surgery, excluding one patient who died 2 weeks after surgery.

**Table 5:** Predictive factors for severe complications or death at the final follow-up period (average 105 months).

Baseline factors	*n*	Hazard ratio	95% CI	*p* value
Age at surgery				
80≤	6	1.22	0.26–5.59	0.80
≤79	31	1.00		
Sex				
Male	23	0.39	0.12–1.23	0.11
Female	14	1.00		
Preop motor JOA (U/E)				
≤2	25	1.41	0.38–5.21	0.60
3≤	12	1.00		
Postop motor JOA (U/E)^*∗*^				
≤2	14	5.64	1.51–21.0	0.01
3≤	22	1.00		
Preop motor JOA (L/E)				
≤1	14	3.16	0.69–14.4	0.13
2≤	23	1.00		
Postop motor JOA (L/E)^*∗*^				
≤1	11	3.47	1.09–10.97	0.03
2≤	25	1.00		

Preop: preoperative; Postop: postoperative; ROM: range of motion; U/E: upper extremities; L/E: lower extremities.

^*∗*^Six months after surgery, excluding one patient who died 2 weeks after surgery.
